# Insights on Metabolic Reprogramming and Its Therapeutic Potential in Acute Leukemia

**DOI:** 10.3390/ijms22168738

**Published:** 2021-08-14

**Authors:** Ludovica Di Martino, Valeria Tosello, Edoardo Peroni, Erich Piovan

**Affiliations:** 1 Dipartimento di Scienze Chirurgiche, Oncologiche e Gastroenterologiche, Universita’ di Padova, 35122 Padova, Italy; ludovica.dimartino@studenti.unipd.it; 2 UOC Immunologia e Diagnostica Molecolare Oncologica, Istituto Oncologico Veneto IOV—IRCCS, 35128 Padova, Italy; valeria.tosello@iov.veneto.it (V.T.); edoardo.peroni@iov.veneto.it (E.P.)

**Keywords:** acute leukemia, targeted therapy, metabolism

## Abstract

Acute leukemias, classified as acute myeloid leukemia and acute lymphoblastic leukemia, represent the most prevalent hematologic tumors in adolescent and young adults. In recent years, new challenges have emerged in order to improve the clinical effectiveness of therapies already in use and reduce their side effects. In particular, in this scenario, metabolic reprogramming plays a key role in tumorigenesis and prognosis, and it contributes to the treatment outcome of acute leukemia. This review summarizes the latest findings regarding the most relevant metabolic pathways contributing to the continuous growth, redox homeostasis, and drug resistance of leukemia cells. We describe the main metabolic deregulations in acute leukemia and evidence vulnerabilities that could be exploited for targeted therapy.

## 1. Introduction

Acute leukemia arises from the malignant transformation of myeloid or lymphoid hematopoietic progenitor cells. Acute myeloid leukemia (AML) is characterized by the accumulation of immature myeloblasts that have a block in differentiation. Acute lymphoblastic leukemia (ALL) originates instead from the transformation of B-cell (B-ALL) or T-cell progenitors (T-ALL). ALL is the most frequent pediatric cancer, representing approximately 80% of childhood leukemias [1]. On the other hand, AML is the most prevalent acute leukemia in adults [2]. Neoplastic transformation involves the acquisition of mutations activating oncogenes and inactivating tumor suppressors, ultimately leading to an aberrant transcriptional and metabolic program that is distinct from their normal cellular counterpart. The connection between cancer cells and modifications in cellular metabolism was first identified almost 100 years ago by Otto Warburg, who proposed the so-called “Warburg effect” [3,4]. Warburg postulated that tumor tissues have increased glucose consumption and lactate production compared to normal tissues, even in the presence of oxygen. While our understanding of tumor metabolism is continuously evolving, altered metabolism is now acknowledged as a hallmark of cancer [5]. Further, tumor metabolism represents a druggable target, thus identifying specific metabolic vulnerabilities within different neoplasia is of much interest. Numerous factors are at the heart of the metabolic rewiring that occurs in tumors (including acute leukemias), amongst which a key role is played by signaling pathways frequently deregulated in cancer such as avian myelocytomatosis viral oncogene homolog (MYC), neurogenic locus notch homolog protein 1 (NOTCH1), Fms Related Receptor Tyrosine Kinase 3 (FLT3), rat sarcoma viral oncogene homolog/mitogen-activated protein kinase (RAS/MAPK), and the phosphoinositide 3-kinase (PI3K)/v-akt murine thymoma viral oncogene homolog (AKT)/mechanistic target of rapamycin (mTOR) pathways [6]. Another aspect to consider in acute leukemias is the peculiar microenvironment in which hematopoietic stem cells (HSCs) reside, which is known to be hypoxic and hypoglycemic, evoking an adaptive response that includes heightened glycolysis over oxidative phosphorylation (OXPHOS) driven by hypoxia-inducible factor-1 (HIF-1) [7] and increased fatty acid oxidation (FAO) due to 5′-AMP-activated protein kinase (AMPK) activation [8,9]. Indeed, an HSC or a more differentiated progenitor is thought to undergo malignant transformation acquiring the capability of indefinite self-renewal, generating leukemia initiating/stem cells (LICs/LSCs) [10]. These cells are able to recapitulate the disease and generate relapse due to their resistance to standard chemotherapies. Interestingly, differently from long-term HSC, LICs are more reliant on OXPHOS, increased redox homeostasis [11], and dependence on amino acid metabolism [12]. These peculiar metabolic dependencies of LICs suggest the future feasibility of selective eradication of LICs by targeting these metabolic vulnerabilities. Knowing and understanding the intricate circuitries leading to dysregulated metabolism downstream of the genetic, transcriptional, and epigenetic changes subsequent to malignant transformation are crucial to identify cancer-specific vulnerabilities. In this review, we discuss the main metabolic pathways dysregulated following leukemia development and which metabolic vulnerabilities may be more beneficial therapeutically.

## 2. Deregulated Metabolic Programs in Leukemia

### 2.1. Glucose Metabolism: Warburg Effect and Metabolic Flexibility

During oncogenesis, cancer cells acquire features that discriminate them from their normal counterparts. One of these features is metabolic reprogramming [13]. Since the 1950s, a key metabolic alteration documented in cancer cells is their reliance on aerobic glycolysis (also known as “Warburg effect”) for energy production instead of OXPHOS [3,4]. This is different from what happens in most nonmalignant cells, which instead rely on OXPHOS to generate energy in the form of adenosine triphosphate (ATP). However, differently from Warburg’s initial observations, numerous studies have refined our knowledge of aerobic glycolysis in cancer cells. In fact, although there is a reduced usage of mitochondria and their electron transport chain (ETC) in cancer cells, this does not mean that they are dysfunctional [14]. Glycolysis converts glucose to pyruvate and yields energy (two molecules of ATP). Finally, pyruvate is converted to lactate during aerobic glycolysis or diverted into the tricarboxylic acid (TCA) cycle, which fuels mitochondrial OXPHOS (much more efficient at producing ATP). Currently, it is thought that this increased glucose usage/consumption in tumor cells is of benefit not so much through energy generation (ATP) but rather because glucose can provide metabolic intermediates necessary to sustain the TCA cycle that are used for the synthesis of nucleotides, amino acids, and lipids [15,16]. Increased glucose uptake and aerobic glycolysis are also present in acute leukemia cells [17], but functional mitochondria and OXPHOS are also fundamental [18]. HSCs are totipotent cells that regulate blood cell numbers. The balance between HSC self-renewal and lineage differentiation is regulated by different factors, including cell metabolism. In fact, in order to maintain their survival and allow them to proliferate/differentiate in the hypoxic bone marrow environment, HSCs adapt their metabolism [7]. The hypoxic environment in which HSCs reside determines the stabilization of HIF alpha subunits (HIF-1α and HIF-2α) due to reduced hydroxylation, ubiquitylation, and proteasomal degradation [19]. This allows HIFα subunits to form functional heterodimers with arylhydrocarbon receptor-nuclear translocator (ARNT) or HIF-1β protein and induces a transcriptional response. More specifically, HIFs promote the upregulation of glycolysis over OXPHOS through the transcriptional activation of glucose transporters (GLUT1) and pyruvate disposal (LDHA and PDK1). Furthermore, HIF-1 increases the expression of pyruvate dehydrogenase kinase (PDK) 2 and 4, which prevent pyruvate entrance in the TCA cycle, and by inhibiting the function of pyruvate dehydrogenase (PDH), HIF-1 impedes its usage for mitochondrial OXPHOS [20]. Another metabolic process that regulates the cell fate of HSCs and contributes to the balance between stemness and commitment is FAO, which is positively regulated by the LKB1–AMPK axis, which controls the transcription of FAO enzymes [8]. Acute leukemias are supposed to arise from HSCs or a more differentiated progenitor, which has acquired the ability to self-renew indefinitely as a result of specific mutations [10]. This cell of origin named LSC/LIC is a rare subpopulation of leukemic cells endowed with leukemia-initiating properties, stemness, and drug resistance. Although (as previously mentioned), increased aerobic glycolysis is a common trait in most AML and ALL cells, the rare LIC population manifests distinct metabolic features (at least in AML) compared to normal HSCs [21]. Indeed, these LICs are dependent on OXPHOS with a lower glycolytic reserve compared to mature (bulk) cancer cells; in addition, they show low levels of reactive oxygen species (ROS) and increased levels of glutathione (GSH), sensitivity to disruption of ETC, lack of glucose dependency for energy production, and reliance on amino acids for OXPHOS metabolism [12]. In AML, this LIC-specific dependency on OXPHOS can be specifically targeted using venetoclax (a BCL2 inhibitor), which suppresses both the TCA cycle and OXPHOS, probably through decreased amino acid uptake, which fuels the TCA cycle in AML LICs [11,12,22]. However, the metabolic plasticity of AML LICs determines an escape from the metabolic pressure of venetoclax treatment through increased FAO that maintains OXPHOS upon amino acid loss. This phenomenon leads to resistance to venetoclax/azacitidine therapy and highlights the importance of including a fatty acid metabolism inhibitor to restore sensitivity to BCL-2 inhibition in AML cells [22]. Interestingly, AML cells show dependence on FAO, even in the absence of treatment exposure, due to low levels of prolyl-hydroxylase 3 (PHD3), which is an activator of acetyl-CoA Carboxylase 2 (ACC2) that normally suppresses FAO. Furthermore, high expression levels of carnitine palmitoyl transferase 1A (CPT1A) and the carnitine transporter CT2 (SLC22A16) also contribute to promoting FAO and constitute therapeutic targets for AML subsets [23,24,25]. Very recently, LICs from relapsed/refractory (R/R) AML patients have been found to be different metabolically from the LICs of de novo AML patients [26]. Indeed, R/R AML LICs have increased nicotinamide levels and metabolism due to increased activation of the rate-limiting enzyme, Nicotinamide Phosphoribosyltransferase (NAMPT), which promotes resistance to venetoclax treatment. Consistently, NAMPT inhibition reduced OXPHOS in R/R LICs by reducing the activity of nicotinamide adenine dinucleotide (NAD)+ dependent TCA enzymes and inhibiting amino acid metabolism and FAO. Interestingly, NAMPT inhibition also targets mature AML blasts through the inhibition of glycolysis, thus representing a novel therapeutic approach for the treatment of R/R AML patients possibly in combination with chemotherapy or venetoclax.

**Figure 1:**
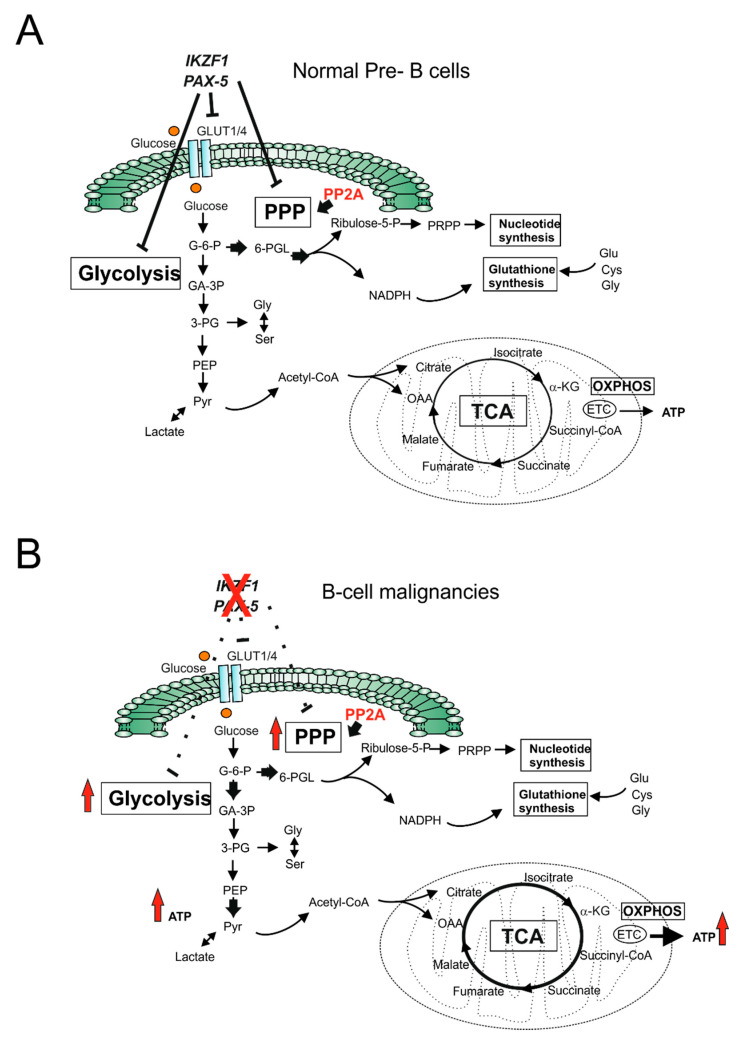
Transcriptional repression of glucose metabolism by metabolic gatekeepers prevents B-cell transformation and is lost during oncogenic signaling. (**A**) Gatekeeper transcription factors (PAX5, IKZF1) limit glucose consumption and the production of metabolites that fuel the tricarboxylic acid cycle (TCA), thus limiting the energy (ATP) production necessary for neoplastic transformation; (**B**) Genetic alterations leading to loss of PAX5 or IKZF1 gatekeeper functions allow an unrestricted consumption of glucose (glycolytic flux) and sufficient energy production to permit transformation. In B cells, protein phosphatase 2A (PP2A) is required in order to further enhance glucose utilization, which is also through the PPP pathway. Solid arrows indicate activity of the pathway; dashed lines indicate inhibited or weak activities. GLUT: glucose transporter; G-6-P: glucose-6-phosphate; GA-3P: glyceraldehyde-3-phosphate; 3-PG: 3-phosphoglycerate; 6-PGL: 6-phosho-gluconolactone; PEP: phosphoenolpyruvate; Pyr: Pyruvate; α-KG: alpha-ketoglutarate; Glu: glutamine; Cys: cysteine; Gly: glycine; Ser: serine; ribulose-5-P: ribulose-5-phosphate; PRPP: phosphoribosyl pyrophosphate; OAA: oxaloacetate; PPP: pentose phosphate pathway; TCA: tricarboxylic acid cycle; OXPHOS: oxidative phosphorylation; ETC: electron transport chain.

Precursor B-ALL (Pre-B-ALL) primary leukemic cells show an upregulation of genes involved in glycolysis associated with a suppression of TCA cycle genes relative to normal CD34+ HSCs [27]. In addition, lactate production is very high even under normoxic conditions, and the inhibition of glycolysis in these cells induces apoptosis, indicating dependence on elevated aerobic glycolysis for survival. Thus, OXPHOS is reduced in these cells, and putative non neutral mutations in subunits of the OXPHOS machinery encoded by mitochondrial DNA (mtDNA) have been described in ALL samples, possibly contributing to altered ETC activity [28,29,30]. The molecular drivers causing the metabolic shift to aerobic glycolysis are diverse and probably act synergistically. The PI3K–AKT–mTOR pathway is one of the most frequently activated signaling pathways in human cancers. Aberrant activation of this signaling pathway in B- and T-ALL can occur by multiple genetic mechanisms, including inactivation of phosphatase and tensin homolog (PTEN), which is a lipid phosphatase that functions as the main negative regulator of the PI3K pathway [31] or more rarely due to gain-of-function mutations in PI3K regulatory and catalytic subunits or in AKT. Furthermore, non-genetic mechanisms for PI3K activation have been described [31], and both lead to increased glycolysis, lipid biogenesis, protein translation, and mitochondrial metabolism [32]. In fact, AKT is a critical driver of the glycolytic phenotype as it stimulates the expression and membrane translocation of glucose transporters, phosphorylates, and increases the enzymatic activity of key enzymes such as hexokinase and phosphofructokinase 2 [33]. AKT also activates mTOR signaling through the inhibitory phosphorylation of tuberous sclerosis 2 (TSC2), which is a negative regulator of mTOR [34]. In breakpoint cluster region-Abelson murine leukemia viral oncogene homolog (BCR-ABL) and mixed-lineage leukemia-ALL1 fused gene from chromosome 9 (MLL-AF9) rearranged leukemia models, the deletion of pyruvate kinase M2 (*Pkm2*) or lactate dehydrogenase A (*Ldha*) in HSCs delays leukemia development, supporting the concept of active glycolysis as an important factor for leukemia initiation [35]. In T-ALL, NOTCH1-activating mutations are present in over 60% of cases and strongly affect the metabolic flux in these cells [36]. NOTCH1 promotes leukemia cell growth through the direct transcriptional regulation of numerous anabolic pathways, including ribosome biosynthesis, protein translation, and nucleotide and amino acid metabolism [37,38]. This pro-oncogenic function is further sustained by the upregulation of MYC, which is a direct NOTCH1 target [39]. Interestingly, although NOTCH1 mutated T-ALL cells depend on aerobic glycolysis, this dependency is less marked than for normal proliferating T cells, which is possibly due to the activation of AMPK by oncogenic NOTCH1 signaling [9]. In fact, AMPK restrains aerobic glycolysis in T-ALL cells by suppressing mTORC1 signaling while augmenting OXPHOS through increased complex 1 activity. Concerning the regulation of glucose metabolism in normal and malignant B-cells, two key B-lymphoid transcription factors paired box gene 5 (PAX5) and IKAROS family zinc finger 1 (IKZF1) act as “metabolic gatekeepers” by transcriptionally repressing glycolytic genes and reducing metabolite flow into the TCA cycle ultimately limiting energy supply [40]. Lesions (deletions, mutations) of these genes are present in a high proportion of pre-B ALL cases, leading to a loss of their gatekeeper functions, resulting in enhanced glycolysis and increased ATP production, favoring malignant transformation (Figure 1). Indeed, PAX5 haploinsufficiency in a BCR-ABL1 transgenic pre-B ALL leukemia model determined increased glucose uptake, glycolysis, and ATP generation. In a further report, the serine/threonine phosphatase 2A (PP2A), which functions as a tumor suppressor in multiple tumor types (due to recurrent deletions of mutations of its subunits) [41], was found to have an essential role in B-cell malignancies [42]. Normal B cells and B-cell malignancies expressing PAX5 and IZF1 have low Pentose Phosphate Pathway (PPP) activity due to the active repression of PPP enzymes (Figure 1). In B-ALL, PP2A shifts glucose utilization from glycolysis to the PPP to mitigate oxidative stress through increased generation of reducing equivalents such as nicotinamide adenine dinucleotide phosphate (NADPH). Loss of PP2A in B-cell leukemia blasts increases glucose utilization and glycolysis products including ATP and lactate, while decreasing PPP-dependent antioxidant protection. These observations identify a gatekeeper function of the PPP in B-cell malignancies amenable for therapeutic intervention through the inhibition of PP2A and glucose-6-phosphate dehydrogenase (G6PD) [42].

### 2.2. One-Carbon Metabolic Network

The term one-carbon (1C) metabolic network identifies a series of biochemical reactions centered around folate compounds that provide one carbon moieties to support multiple biological functions critical for cell growth and survival. Through the cytosol and mitochondria, a complex network of metabolic enzymes supports the synthesis of nucleotides, certain amino acids, and GSH, as well as other cellular processes important for the rapid proliferation of malignant cells [43]. In the following paragraphs, we will describe the main aspects and enzymes that characterize this network in order to identify new therapeutic strategies for the treatment of acute leukemia. Amongst the earliest anti-cancer drugs are antimetabolites, which target metabolic pathways supporting nucleotide biosynthesis (1C metabolism). These include antifolates, such as methotrexate (MTX), which inhibits dihydrofolate reductase (DHFR), thus compromising thymidylate and purine synthesis [44] and 5-fluorouracil, which targets thymidylate synthetase (TYMS) responsible for converting deoxyuridine monophosphate (dUMP) into deoxythymidine monophosphate (dTMP), thereby blocking DNA replication. However, other enzymes (discussed below) involved in this network could represent good candidates for the development of targeted therapies both in ALL and AML.

#### 2.2.1. Folate Cycle: What Have We Learned from the Use of Methotrexate in Leukemia?

Folate, also known as folic acid, is the generic term to identify water-soluble vitamin B9. Inside the cells, folic acid is firstly reduced by DHFR to dihydrofolate (DHF) and finally to tetrahydrofolate (THF). Then, THF is polyglutamated by the enzyme folyl-polyglutamate synthetase (FPGS) [45]. THF is the leading carrier of formaldehyde, and once transferred, the key intermediate 5,10-methylene-tetrahydrofolate (5,10-methylene-THF) initiates the one-carbon cycle [46]. This cycle involves different THF derivatives that take part in the de novo mitochondrial thymidylate biosynthesis pathway as well as de novo glycine and purine synthesis [47]. The high rate of proliferation of cancer cells is a peculiar feature that makes them susceptible to a lack of nucleotides, leading to their death due to the inability to synthesize DNA. Proof of principle for the central role played by the 1C network in sustaining processes critical for neoplastic cell proliferation and growth came from the use of aminopterin (a folate inhibitor of nucleotide biosynthesis) in the clinic, where it induced remission in children with ALL [48]. MTX is an analog of aminopterin that acts as a competitive inhibitor of DHFR to disrupt the folate cycle and causes apoptosis [49]. High-dose MTX is used in maintenance therapy in combination with other chemotherapeutic drugs in patients with ALL [50]. MTX enters cells through the solute carrier family 19 member 1 (SLC19A1) transporter, gets polyglutamated by FPGS, and inhibits DHFR with high affinity [51]. MTX also inhibits the TYMS enzyme, which is necessary for the synthesis of thymidine nucleotides [52]. The action of MTX is limited by the intervention of ATP-binding cassette proteins (ABCC1-4 and ABCG2), which export MTX out of cells. Hematological cancer cell lines show high sensitivity to this antifolate compared to other cancer cell lines. Indeed, ALL and AML cell lines display the highest sensitivity to its cytotoxic effects [53]. However, MTX lacks specificity and has considerable undesirable cytotoxicity [54]. Indeed, DHRF and TYMS enzymes are expressed in many proliferating tissues, which are thus also exposed to the cytotoxic effects of MTX [55].

Thus, it is not surprising that the response to MTX therapy is highly dependent on the activity of the key enzymes and transporters involved in the folate/MTX metabolic pathway. Increased levels of DHFR compromise the antileukemic activity of MTX in childhood ALL, while higher intracellular levels of MTX with long glutamate chains, correlated with higher FPGS activity, are associated with improved survival in ALL patients [56,57]. Interestingly, the amount and length of the polyglutamate chains on MTX are key determinants of antifolate-mediated cytotoxicity. In fact, blasts from AML patients are less sensitive to MTX compared to blasts from ALL patients, which is due to poorer polyglutamation and reduced amounts of MTX within cells (due to a lower activity of FPGS and increased expression of ABC efflux transporters) [58]. Furthermore, B-ALL blasts express higher levels and enzymatic activity of FPGS, rendering these cells more sensitive to MTX compared to T-ALL blasts, which instead show higher levels of DHFR and TYMS [59]. Although pharmacological approaches have been attempted to discover genetic markers involved with MTX toxicity and therapy response, none have been unequivocally identified [60].

#### 2.2.2. Mitochondrial Targeting of the 1C Network as a New Therapeutic Strategy in Acute Leukemia

Folate metabolism occurs in parallel in the cytosol and in mitocondria, which is a feature that makes this metabolism appealing for selective therapeutic intervention. The main source of 1C units is serine (Ser), which is used as a substrate by serine hydroxymethyl transferase, SHMT1 (cytoplasmatic) and SHMT2 (mitochondrial), which leads to glycine and 5,10-methylene-THF (methylene-THF) by transferring the 1C unit to THF [61]. The subsequent enzymatic steps of the folate cycle involve the transfer of the carbon unit between different forms of THFs. In the cytoplasm, a trifunctional enzyme named methylenetetrahydrofolate dehydrogenase 1 (MTHFD1) interconverts methylene-THF to 10-formyl-tetrahydrofolate (10-formyl-THF). This enzyme contains three domains: methylenetetrahydrofolate dehydrogenase, cyclohydrolase, and formyltetrahydrofolate synthetase domains. In the mitochondria, the production of 10-formyl-THF is carried out by two MTHFD isozymes, MTHFD2 and MTHFD2-like (MTHFD2L), via two steps. MTHFD2 with its dehydrogenase activity converts methylene-THF to 5,10-methenyl-tetrahydrofolate (methenyl-THF), which is finally converted in 10-formyl-THF by the cyclohydrolase domain [62]. A potential target within the folate mitochondrial cycle is MTHFD2, which is normally expressed during embryonic development and is almost undetectable in adult tissues. Interestingly, among 1454 metabolic enzymes differentially expressed in cancer tissues, enzymes involved in the folate pathway were found to be amongst the most differentially expressed genes between cancers and normal cells, with MTHFD2 ranking at the top of the list [55]. Although MTHFD2L expression levels in AML are higher than normal tissues when compared to MTHFD2 levels, MTHFD2 seems a more attractive therapeutic target due to the fact that MTHFD2L is unable to compensate the loss of MTHFD2. In particular, MTHFD2 suppression results in impaired growth and induces differentiation in AML cell lines and primary AML blasts. Moreover, the response to MTHFD2 suppression seemed to be higher for those AML patients with FMS-like tyrosine kinase 3 (FLT3)-internal tandem duplication (ITD) mutations. This suggests that patients with FLT3-ITD mutations may benefit from a combination treatment targeting FLT3 and MTHFD2 [63].

Another mitochondrial enzyme extensively studied in recent years is SHMT2. In fact, it seems that targeting this enzyme may improve the response to MTX [64]. Indeed, the use of a dual inhibitor of SHMT1/2, SHIN2, was able to inhibit proliferation in vitro and exert anti-leukemic effects in vivo as a single agent in models of T-ALL (NOTCH1-driven T-ALL mouse models and patient-derived T-ALL xenografts). Interestingly, this treatment was shown to synergize with MTX both in vitro and in vivo, especially in MTX-resistant human T-ALL cells. Importantly, several studies have evidenced the need of dual inhibition for obtaining anti-proliferative effects, due to redundancy between SHMT1 and SHMT2 enzymes. Altogether, these findings suggest that the inhibition of SHMT enzymes synergizes with MTX because MTX depletes THF within cells that represents the substrate for SHMT enzymes [65]. Given these observations, it seems that SHMT1/2 and MTHFD2 could be promising targets within the folate metabolism in acute leukemia and encourage combinations between SHMT1/2 or MTHFD2 inhibitors and drugs targeting upstream enzymes such as DHFR. This may be due to the fact that these enzymes are a crucial part of the THF cycle in the mitochondria and interference with their activities creates a bottleneck in the reaction cycle.

### 2.3. Amino Acid Metabolism and Therapeutic Opportunities in Acute Leukemia:

#### 2.3.1. Serine Synthesis Pathway

Ser is a non-essential amino acid with a key role in several cellular processes. Ser-derived 1C units are required to generate important precursors for the synthesis of lipids, nucleic acids, and other cofactors [66]. Exogenous Ser is crucial for the biosynthetic processes of many cancer cells [67], while others depend primarily on de novo Ser synthesis for growth and survival [68,69]. De novo synthesis proceeds from 3-phosphoglycerate (3-PG), an intermediate product of glycolysis, and three sequential enzymatic reactions lead to the formation of Ser [70]. First, the enzyme phosphoglycerate dehydrogenase (PHGDH) oxidizes 3-PG to 3-phosphohydroxypyruvate (3-PHP), which is subsequently converted by phosphoserine aminotransferase (PSAT1) in 3-phosphoserine (3-PS). Finally, the 3-PS is dephosphorylated to Ser by 1-3-phosphoserine phosphatase (PSPH) [71]. By regulating the expression of PHGDH, PSAT, PSPH, and other metabolic enzymes in the serine synthesis pathway (SSP), cancer cells catabolize Ser to sustain their survival. Amplifications of the gene encoding PHGDH have been reported to support tumorigenesis in melanoma and breast cancer cell lines [72,73]. In a subgroup of patients with lung cancer, high levels of PHGDH have been associated with poor prognosis [74]. Notably, a high expression of PSPH correlates with disease progression and mortality in patients with hepatocellular carcinoma, indicating that the PSPH protein is a potential prognostic biomarker for this cancer [75]. These findings point out the important role of Ser synthesis in the metabolic reprogramming of cancer cells, suggesting that it could represent a valuable therapeutic target.

In T-ALL, the genes involved in Ser biosynthesis have been found to be upregulated, in particular the PSPH enzyme. Specifically, the introduction of the ribosomal mutation RPL10 R98S drives cells to become more dependent on Ser synthesis for purine biosynthesis. Interestingly, Ser catabolism promotes the release of glycine from cells (a product of this pathway), which promotes proliferation and survival of the surrounding cells. Furthermore, inhibiting the expression of PSPH in T-ALL cell lines has been found to induce cytostatic effects in vitro and reduce leukemia expansion in vivo. Although the mechanisms that render T-ALL cells vulnerable to inhibition of this pathway are still unknown, these results demonstrate that the targeting of PSPH may serve as a novel therapeutic approach for a subgroup of T-ALL patients [76].

The flux through 1C metabolism must remain plastic for cells to regulate levels of the related nutrients in response to changing intra- and extracellular conditions. Some leukemic cells lines (HL-60, K-562, and THP-1) resist glutamine (Gln) deprivation by regulating enzymes such as PHGDH and PSAT in order to maintain viability. In this context, the catabolism of Ser leads mainly to the production of GSH, which is able to antagonize the ROS produced in excess due to the lack of Gln [77]. Consequently, inhibition of the enzymes part of the SSP may be effective in increasing the efficacy of Gln targeting therapies.

The ability of some cancer cells to increase their growth by increasing fructose uptake as a compensation strategy in the presence of limited glucose availability is another adaptive mechanism. Indeed, in a recent study [78], AML cells in fructose-rich conditions were found to become dependent on Ser biosynthesis for tumor growth. More specifically, they demonstrated that under fructose-rich conditions, there is an increase of the nicotinamide adenine dinucleotide (NAD+)/NADH ratio, which leads, via a higher Ser synthesis flux, to the generation of α-KG from Gln, ultimately facilitating TCA anaplerosis. Furthermore, using an in vivo model that recapitulated a fructose-rich tumor microenvironment, they demonstrated that PHGDH supports leukemogenesis, and its inhibition could be a novel therapeutic option for patients with leukemia, especially with high levels of fructose in the bone marrow.

In conclusion, these results, although still preliminary, encourage the targeting of Ser metabolism in leukemia. Further work is required to determine the contexts in which SSP flux and/or consumption of extracellular Ser are necessary for leukemic growth.

#### 2.3.2. Metabolic Dependencies as a Result of an Increased Demand for Glutamine

Gln is considered as the most abundant amino acid in the body; in fact, its concentration in plasma is between 10 and 100-fold in excess with respect to other amino acids, ranging from 500 to 800 μM/L. Lymphocytes, macrophages, and in general cells of the immune system have high rates of Gln consumption, making Gln supplementation a crucial step to support patients with an immune suppressive status [79]. Gln is also important for cancer cells, both for hematological and solid tumors, and its metabolism has been investigated as a therapeutic target in different tumor types [80,81]. Gln is not only used for the biosynthesis of fundamental building blocks such as proteins and nucleotides but also for energy production. In fact, Gln is converted via the enzyme glutaminase (GLS) to glutamate (Glu), which is in turn converted to α-ketoglutarate (α-KG) by glutamate dehydrogenase (GLUD1) or transaminated by glutamate-linked transaminases. α-KG can enter in the TCA or can be used for the production of GSH, which is particularly important for cancer cells that are exposed to increased ROS production (recently reviewed in [82]). Gln is a non-essential amino acid, and its cellular content is normally regulated both by influx from the extracellular milieu (via multiple transporters) and by the conversion of Glu and ammonia to Gln catalyzed by glutamine synthetase (GS). Cancer cells are often addicted to Gln because this reaction (Gln synthesis) is not sufficient to satisfy the increased demands of highly proliferative cells, making these cells highly dependent on exogenous Gln for cell growth and survival. The overexpression of SLC1A5 (also known ASCT2), a sodium-dependent transporter for neutral amino acids with high affinity for Gln, is highly expressed in several cancer types, especially those with MYC overexpression. Indeed, c-MYC has been demonstrated to be a key regulator of ASCT2 and GLS in cancer, thus promoting Gln uptake and glutaminolysis [83,84]. In AML cells, ASCT2 knockdown induces apoptosis and inhibits tumor formation in a mouse AML xenograft model [85]. The role of ASCT2 in Gln and amino acid metabolism has been recently studied both in normal and malignant HSCs using a conditional mouse model where *Slc1a5* was deleted in the germline [86]. These mice have a normal lifespan but show mild alterations of hematopoiesis, leading to lower white blood cell counts and a reduction in the frequency and absolute number of HSCs. On the contrary, *Slc1a5* deletion causes a reduction in AML development in mouse models of leukemia induced by the overexpression of *MLL-AF9* oncogene or following *Pten* deletion. Mechanistically, the loss of *Slc1a5* induces a global effect on metabolism, reducing leucine influx and mTOR signaling and ultimately leading to apoptosis in leukemia cells. Notably, a variant of ASCT2 with an N-terminal targeting signal for mitochondrial localization has been recently identified in pancreatic cancer cells [87]. This variant contributes to metabolic reprogramming promoting ATP generation, GSH synthesis, and drug resistance in cancer cells. Importantly, this variant is induced by low oxygen tension, contributing to drug resistance in hypoxic niches. Another crucial step in Gln metabolism is the initial reaction that converts Gln to Glu via GLS, which is often de-regulated in cancer. Two isozymes of glutaminase exist: a kidney-type (GLS) and a liver-type enzyme (GLS2 or LGA) [88]. GLS has been intensively studied, as it has been linked to the progression of a number of cancers and thus targeted by extensive drug discovery efforts (recently reviewed in [89]). In AML, the GLS inhibitor, CB-839, inhibits GSH production, induces mitochondrial ROS (mitoROS), and determines cell death [90]. In addition, combination treatments including GLS inhibitor and drugs that induce mitoROS have shown to be effective therapeutically both in vitro and in vivo in AML and B-ALL models. Other preclinical studies demonstrate the efficacy of CB-839 in AML cells, especially those harboring specific genetic defects such as isocitrate dehydrogenase (IDH)-mutated AML cells [91] or in combination with other drugs, such as the BH3 mimetic ABT-199, which specifically inhibits BCL-2 [92]. Glutaminolysis is also crucial in NOTCH1-induced T-ALL cells where Gln is converted to Glu and incorporated in the TCA cycle, contributing to a high percentage of TCA cycle intermediates. Indeed, the inhibition of glutaminolysis using a specific inhibitor (BPTES) synergistically enhances the anti-leukemic effects of anti-NOTCH1 therapy in a mouse model of T-ALL [93]. Furthermore, the pharmacological inhibition of WEE1, a nuclear tyrosine kinase involved in cell cycle checkpoint signaling, leads to dependency on glutaminolysis for survival, making the dual targeting of WEE1 and GLS synergistic lethal in T-ALL [94]. Similarly, in AML cells, glutaminolysis becomes a therapeutically targetable vulnerability when combined with specific tyrosine kinase inhibitors in FLT3-ITD-driven leukemias [95]. Metabolic vulnerabilities acquired under the pressure of first-line chemotherapy can also affect the outcome of patients with leukemia. In fact, particularly in AML, remission is often achieved but unfortunately, in the majority of patients, a few resistant cells persist (LICs), which are responsible for relapse. Recently, an elegant study analyzed the metabolic profile of residual cells that survive after chemotherapy in their native environment and found that persisting cells upregulated Gln metabolism [96]. Surprisingly, residual cells used Gln to fuel GSH synthesis and pyrimidine metabolism. In particular, these persisting cells were particularly sensitive to a time-specific inhibition of pyrimidine synthesis, which was supported by leptin receptor-positive stromal cells that provided aspartate as a substrate for this reaction. In conclusion, many preclinical studies confirm the efficacy of targeting Gln metabolism, and further efforts are necessary to translate these observations to the clinic. Currently, in fact, only one clinical trial is ongoing in high-risk myelodysplastic syndrome (MDS) patients combining azacitidine (a hypomethylating agent) with CB-839 (Phase I/II, NCT03047993) [97].

#### 2.3.3. Impaired Synthesis of Asparagine as a Metabolic Vulnerability

Asparagine (Asn) is a non-essential amino acid in humans, which can be synthesized by cells from metabolic intermediates. A key enzyme in the synthesis of Asn is asparagine synthetase (ASNS), which transforms aspartate into Asn and Gln to Glu. Asn is primarily involved in protein synthesis, and its residues can provide a site for glycosylation in the carbohydrate chain to form the N-linkage [98]. Asn is also important for cancer cells where Asn plays an important role as an amino acid exchange factor, modulating the concentration of other amino acids, especially Ser, arginine (Arg), and histidine (His), and consequently crucial for activating mTORC1 and promoting cell growth [99]. Importantly, acute leukemia cells are auxotrophic for Asn because they express low levels of ASNS, and this phenotype leads to high sensitivity to asparaginase. The relevance of Asn metabolism for ALL cells was first discovered when the serum of guinea pigs was found to specifically cause significant regression of engrafted mouse lymphomas [100,101]. This intuition was subsequently confirmed by others [102,103], who identified asparaginase as the serum component that determined the phenotype. L-Asparaginase (ASNase) is now standard of care for the treatment of ALL in combination with other chemotherapeutic drugs and is considered an essential drug by the World Health Organization. Clinical-grade ASNase, approved for ALL treatment, comes from two different species of bacteria, Escherichia coli (E coli ASNase) and Erwinia chrysanthemi (Erw ASNase), which are both quite effective. First-line treatment consists of polyethylene glycol conjugated E coli ASNase due to its lower immunogenicity, while patients hypersensitive to E coli ASNase can switch to Erw ASNase. Both ASNases are able to deplete Asn and Gln (they possess GLS activity) [104]. In recent years, a huge effort has been made to develop novel ASNase drugs in order to reduce hypersensitivity, side effects, and to modulate activity for Gln (reviewed in [105]). The identification of new drugs should also solve problems related to Erw ASNase availability that caused a discontinuation of ASNase doses, lowering disease-free survival in high-risk ALL patients [106]. The mechanisms that control ASNS expression and its correlation with ASNase sensitivity are very complex and not completely elucidated. In B-ALL and T-ALL cell lines, ASNase sensitivity was initially correlated with ASNS protein levels but not its mRNA levels [107]. A more complex situation was described by another study using both ALL cell lines and diagnostic ALL samples. Specifically, ALL blasts, differently from ALL cell lines, express very low levels of ASNS and correlation between its expression and sensitivity to ASNase was not informative [108]. Recently, methylation of CpG islands in ASNS gene has been demonstrated as one of the epigenetic mechanisms responsible for ASNS gene silencing in B-ALL; higher ASNS methylation is described to be associated with higher ASNase sensitivity through lower ASNS transcript and protein levels [109]. In T-ALL, T-cell leukemia homeobox protein 1-positive (TLX1+) patients express low levels of ASNS when compared with TLX3+ and TLX-negative patients, due to epigenetic silencing of ASNS by both DNA methylation and a decrease of active histone marks; in addition, promoter methylation of the ASNS gene correlated with ASNase sensitivity in both T-ALL cell lines and patient-derived xenografts [110]. One major problem in ASNase treatment is represented by the development of resistance associated to poor prognosis, leaving patients without alternative therapeutic options. The concept that elevated ASNS expression can induce ASNase resistance was developed by researchers some time ago [111,112]. However, ASNS expression is not the only factor that contributes to ASNase response, and other mechanisms need to be further elucidated to improve prognosis in acute leukemia patients [113,114]. In this context, using a CRISPR-based genetic screen, a crucial role for activating transcription factor 4 (ATF4) in the adaptive response to amino acid deprivation was found in T-ALL cells [115], supporting previous observations [116]. In addition, this study showed that the deprivation of Asn specifically determined a significant induction of zinc finger and BTB domain containing 1 (ZBTB1), which is a transcription factor involved in T cell development [117,118], whose loss was sufficient to sensitize resistant T-ALL cells to ASNase both in vitro and in vivo. ASNase resistance was also recently associated with huntingtin associated protein 1 (HAP1) expression in ALL cells through an experimental approached based on an unbiased genome-wide RNA interference screen [119]. Mechanistically, HAP1 interacts with huntingtin and the intracellular Ca^2+^ channel, inositol 1,4,5-triphosphate receptor (IP3R), to form a ternary complex that mediates endoplasmic reticulum (ER) Ca^2+^ release upon stimulation with inositol 1,4,5-triphosphate (IP3). Loss of HAP1 prevents the formation of the ternary complex and thus ASNase-mediated ER Ca^2+^ release and apoptosis. In another relevant study, a genetic screen revealed the synthetic lethality of Wnt activation and ASNase [120]. According to this study, Wnt signaling induces ASNase sensitivity by inhibiting glycogen synthase kinase 3 (GSK-3)-dependent protein degradation (a catabolic source of Asp); moreover, GSK-3α inhibition was able alone to induce sensitization to ASNase similarly to the activation of Wnt signaling. Importantly, combination therapies targeting both Asn synthesis and GSK-3α were able to overcome resistance in leukemic cells. In conclusion, many advances have focused on the characterization of new forms of ASNase that may increase the bioavailability, efficacy, and present reduced side effects. In parallel, new important findings will support combination therapies with the final aim of killing ASNase-resistant cells.

#### 2.3.4. Emerging Roles of Tryptophan Metabolism

Tryptophan (Trp) is a non-polar aromatic and essential amino acid in humans. Although a small portion of Trp is used for protein biosynthesis and neurotransmitter production, the majority of the Trp pool is degraded in the kynurenine pathway (KP), which generates several metabolites mainly involved in immunomodulation. The first step of the KP is tightly regulated and consists of the conversion of Trp by the indoleamine-2,3-dioxygenase enzymes (IDO1 and IDO2) and tryptophan-2,3-dioxygenase (TDO) in N-formylkynurenine (NFK), which is subsequently converted into kynurenine (KYN), which is a central metabolite in KP [121]. High levels of IDO1 have been found in several tumors in association with cancer progression and poor prognosis [122]. Several studies support an important role for IDO1 in creating a favorable environment for tumor cells by inhibiting mature T-cell proliferation and inducting regulatory T cells [123,124,125,126]. In addition to IDO1, high levels of KYN are implicated in cancer progression. In a recent study centered on colon cancer, the KYN levels in tumor cells were higher than in adjacent normal cells due to upregulation of the specific transporter SLC7A5 and the enzyme arylformamidase (AFMID), which is involved in the conversion of Trp into KYN [127]. High levels of KYN can interfere with oncogenic signaling pathways, such as PI3K-AKT, promoting cellular proliferation and resistance to apoptosis [128]. Importantly, repopulating tumor cells can escape immune surveillance by transferring to adjacent CD8+ T cells the KYN metabolite, which in turn activates the aryl hydrocarbon receptor (AhR) and increases PD-1 expression [129]. The expression levels of IDO1 and KYN and the activity of KP have also been investigated in the context of hematological malignancies, in particular in AML, where an extensive review of the literature has been recently performed [130,131]. Briefly, an initial study found that the KYN/Trp ratio was higher in the serum of AML patients compared to healthy controls, suggesting that increased IDO activity is associated with lower survival [132]. Subsequent studies showed that serum KYN levels alone [133] or in combination with expression levels of IDO1 [134] can have an important prognostic role in AML and also in other hematological malignancies such as adult T-cell leukemia/lymphoma [135,136], peripheral T-cell lymphoma [137] and in diffuse large B-cell lymphoma [138]. Similar results have also been obtained analyzing IDO1 expression in AML blasts at the transcript level [139,140] or at the protein level using immunohistochemistry [141]. The importance of KP in different diseases has promoted the development of IDO and TDO inhibitors and modulators [142]. The IDO inhibitor, 1-methyl-tryptophan (1-MT), has been shown to be effective in reverting the generation of regulatory T-cells both in vitro and in vivo in leukemia models [143]. Due to the development of multiple inhibitors, there are now several active clinical trials, especially in solid tumors, where they are used in combination with chemotherapy or checkpoint inhibitors, with encouraging results [122]. In the context of hematological malignancies, initial clinical trials have been performed not only in AML patients but also in MDS patients, where KP activation has been demonstrated [144]. In fact, a phase 2 clinical trial in MDS patients showed minimal toxicity with no patient undergoing progression when using the potent IDO1 inhibitor, epacadostat (NCT01822691). However, median overall survival was not calculated given the reduced number of participants, and new trials are needed to better understand the efficacy of this drug in MDS. In addition, a phase 1 clinical trial on AML patients using indoximod, another IDO1 inhibitor, in combination with chemotherapy has been recently concluded (NCT02835729). The recent data obtained from preclinical studies and clinical trials are encouraging. Further studies using IDO inhibitors in combination with other drugs, such as checkpoint inhibitors, will probably open new therapeutic opportunities in AML and MDS patients in the near future.

#### 2.3.5. Branched Chain Amino Acid (BCAA) Metabolism: An Emerging Oncogenic Pathway

Among the nine essential amino acids, leucine, isoleucine, and valine are the most abundant components in proteins [145]. These amino acids, also known as branched chain amino acids (BCAAs), promote protein synthesis and represent sources of nitrogen and carbon for biosynthetic processes and energy production [146]. BCAAs levels are tightly controlled: they can be transaminated by BCAAs transaminase (BCATs) enzymes and further oxidized by the branched chain α-keto acid dehydrogenase enzyme complex (BCKDC). The BCAT enzymes, encoded by two different genes, are localized either in the cytosol (BCAT1) or in the mitochondrion (BCAT2). These enzymes convert BCAAs into their corresponding branched-chain α-keto acids (BCKAs) by transferring the amino group onto α-KG and thereby generating Glu. This is a reversible reaction, enabling the production of BCAAs by reamination of BCKAs possibly coming from other tissues [147]. BCAAs play an important role in sustaining T-cell activation [148]. Specifically, this is mainly associated with the known role of leucine as an activator of mTORC1, which is a master regulator of cellular processes that senses energy and nutrients [149]. Importantly, based on transporter and BCAT1/2 activities, the uptake and metabolism of BCAAs is finely regulated. In Foxp3^+^ Treg cells, the isoleucine-SLC3A2 transporter axis directly promotes the mTOR pathway activation necessary to control their function and maintenance in the periphery [150]. Following T-cell receptor (TCR) engagement, activated T cells trigger the expression of SLC7A5 and BCAT1 to promote leucine uptake and cytosolic transamination, respectively. In fact, although leucine directly controls the activity of mTORC1 necessary for the activation of T cells, the induction of BCAT1 following TCR engagement limits leucine availability to prevent the hyperactivation of T cells [151].

In the past few years, the metabolism of BCAAs has received significant scientific interest, particularly given its association with specific cancer phenotypes. Notably, changes in BCAAs metabolism in different cancers are largely due to the overexpression of BCAT1. In fact, in contrast to BCAT2 (almost ubiquitously expressed), BCAT1 has limited expression in adult tissues (except for the nervous system and activated T cells), while it is often expressed in many tumors such as gliomas, ovarian cancer, non-small lung carcinoma, and myeloid leukemia [146]. In these diseases, BCAT1 could represent an important prognostic cancer marker [147]. Recent findings highlight a key role for BCAT1 in acute leukemia in the control of BCAAs, especially at the level of LSCs/LICs, paving the way for new therapeutic strategies.

**Figure 2:**
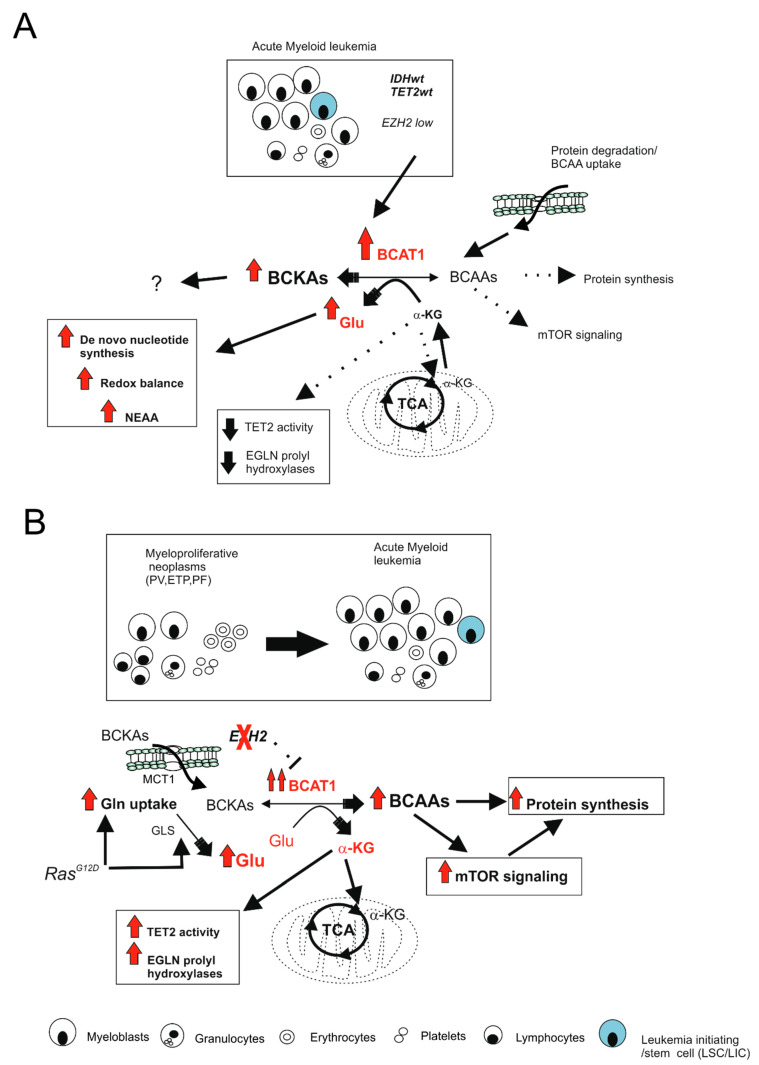
Oncogenic functions of BCAT1 are highly dependent on mutational and cellular context. (**A**) In AML, LICs showing high expression of BCAT1 but low levels of EZH2 (IDH1/2wt, TET2wt, EZH2wt genotype), BCAT1 catalyzes BCAA deamination with depletion of α-KG. This leads to a reduced activity of α-KG-dependent histone and DNA demethylases and α-KG-dependent dioxygenases with DNA hypermethylation and stabilization of HIF-1α; (**B**) In the transition from myeloproliferative diseases (MPN; PV, ETP, PF) to acute leukemia, EZH2 loss reactivates BCAT1 expression, while oncogenic RAS (NrasG12D) increases glutamine (Gln) uptake and consumption. This leads to enhanced BCAT1-catalyzed BCKA reamination to BCAAs and subsequent mTORC1 activation. The activity of α-KG-dependent enzymes is also enhanced as well as Glu-dependent processes such as nucleotide synthesis, redox balance, and protein synthesis. All these events contribute to myeloid transformation. Solid arrows indicate the activity of the pathway, dashed lines indicate inhibited or weak activities. GLS: glutaminase; Gln: glutamine; Glu: glutamate; BCAAs: branched chain amino acids; BCKA: branched chain α-keto acids; α-KG: apha-ketoglutarate; TET2: Ten-eleven translocation 2 demethylase; mTOR: Mammalian target of rapamycin; NEAA: non-essential amino acids; LICs: leukemia-initiating cells; PV: polycythemia vera; ETP: essential thrombocytopenia; PF: primary myelofibrosis.

Different cellular processes such as gene expression, cell-cycle progression, and DNA repair are controlled by epigenetic mechanisms that in turn can be regulated by metabolic changes. In AML, BCAAs metabolism interferes with the activity of multiple α-KG-dependent histone and DNA demethylases, including the Ten-eleven translocation (TET) and Jumonji domain-containing histone demethylase (JHDM) family of demethylases. More specifically, increased BCAT1 activity/expression found in AML patients (with isocitrate dehydrogenase 1 (IDH1) and TET2 wild-type (wt) status) determines a reduction in α-KG levels due to the transfer of nitrogen from BCAAs to α-KG, resulting in a loss of TET and JHDM activity. This leads to DNA hypermethylation in AML with high BCAT1 expression. Moreover, the reduced levels of α-KG also affect the activity of α-KG-dependent dioxygenases, such as EGLN prolyl hydroxylases, which catalyze hydroxylation on proline residues of HIF-1α, leading to its stabilization under normoxia (Figure 2A). Together, these reprogramming events (which mimic IDH1/2 mutations; see below) affect AML progression by influencing gene transcription and preventing differentiation [152]. Recent evidence suggests that BCAT1 is not only able to control the epigenetic state of cells by means of its activity, but it in turn comes to be controlled epigenetically contributing to neoplastic transformation. In fact, a recent study demonstrated that mutations in *Ras*, such as the G12D mutation, together with loss of function mutations of Enhancer of zeste homolog 2 (*Ezh2)* cooperate to induce leukemic transformation in mice with myeloproliferative neoplasms (MPN) due to epigenetic and metabolic reprogramming. Notably, in this mutational landscape, BCAA metabolism, TCA cycle, and mTORC1 signaling were the main metabolic pathways involved in leukemic transformation with BCAT1 reactivation occurring in NrasG12D^+/−^Ezh2^−/−^ LICs. In HSC and progenitor cells, the BCAT1 gene is physiologically silenced by the H3K27me3 repressive mark on its promoter. Conversely, in human AML with low expression of EZH2, the decrease in H3K27me3 marks reactivates BCAT1 expression and BCAAs metabolism. More specifically, in NrasG12D^+/−^Ezh2^−/−^ LICs, the MCT1 transporter increases the uptake of BCKAs that are used by BCAT1 to produce BCAAs, which in turn induced the activation of mTORC1 to sustain protein synthesis (Figure 2B). Thus, in NrasG12D^+/−^Ezh2^−/−^ mice, the inhibition of BCAT1, MCT1, and GLS resulted in a suppression of mTORC1 activity together with a reduction in tumor burden and increased survival [153].

Altogether, these findings reveal a key role for BCAT1 in the process of leukemogenesis/tumorigenesis and bring to the forefront the importance of cellular context. Indeed, although increased BCAT1 expression has been found in numerous tumors and associated with clinical aggressiveness, distinct roles for this transaminase have been proposed. These different mechanisms are related to whether BCAT1 catalyzes BCAA deamination with Glu production (and α-KG depletion) or BCKA reamination, resulting in increased BCAA and mTOR activation. In glioblastomas, those carrying wild-type IDH1/2 show BCAT1 expression. Here, BCAT1 is activated to determine BCAA deamination and Glu production. IDH-mutant gliomas convert isocitrate into 2-hydroxyglutarate, which competitively inhibits α-KG-dependent DNA and histone demethylases, contributing to the downregulation of BCAT1/2 function through DNA methylation of its promoter [154]. Non-small lung cancer tumors increase BCAA uptake as a nitrogen source and incorporate them into tissue proteins [155].

Summarizing, in the context of hematological tumors, AML LICs (with IDH1/2wt, TET2wt, EZH2wt genotype) present high levels of BCAT1, which catalyzes BCAA degradation and the depletion of α-KG, which reduces the activity of demethylases such as TET2 and prolyl hydroxylases such as EGLN1 (leading to DNA hypermethylation and stabilization of HIF-1α). Thus, this occurs in the absence of TET2 and IDH mutations. Meanwhile, in the MPN-to-leukemia transformation (and possibly AML cases presenting with activating *RAS* mutations and *EZH2*-deficiency), activated BCAT1 cooperates with increased Gln uptake to enhance BCKA reamination, leading to increased BCAAs and mTORC1 signaling (Figure 2). In conclusion, although BCAA metabolism may represent a therapeutic target in both MPNs and AMLs, the mechanisms behind the oncogenic function of BCAT1 may be highly dependent on mutational and cellular context.

## 3. Conclusions

Targeting tumor metabolism is an attractive strategy because the metabolic heterogeneity within and between tumors is reduced compared to the genetic heterogeneity of tumors, which is often unique. In fact, only a minority of patients can access personalized precision therapy based on their genomic profile. The studies discussed in this review evidence the evolution of our understanding of the metabolic dysregulation and rewiring of established leukemia; however, the metabolic programs underlying leukemia initiation and progression remain to be elucidated. Nevertheless, targeting tumor metabolism poses numerous challenges, as tumors often have multiple hyperactive metabolic pathways and show considerable metabolic plasticity following nutrient deprivation or the selective inhibition of some pathways. This may be particularly relevant for leukemias sustained by LICs. In addition, the interaction of leukemic cells with the bone marrow microenvironment should also be considered in the development of therapeutic strategies. Further in-depth investigation, using improved analytical techniques (for analysis of small cell numbers typically available in the clinical setting), on identifying the metabolic dependencies of de novo and R/R LICs or across the various genetic and molecular leukemia subgroups will increase our understanding of metabolic dependencies and survival strategy of leukemia cells. In conclusion, although numerous exciting drug combinations have been proposed in this review, it must be mentioned that many are not ready for clinical deployment, and it would be highly desirable to conduct clinical trials that include predictive markers of response to drugs targeting tumor metabolism to demonstrate an on-target effect.

## Data Availability

Not applicable.
